# Adaptive list sequential sampling method for population-based observational studies

**DOI:** 10.1186/1471-2288-14-81

**Published:** 2014-06-25

**Authors:** Michel H Hof, Anita CJ Ravelli, Aeilko H Zwinderman

**Affiliations:** 1 Department of Clinical Epidemiology, Bioinformatics, and Biostatistics, Academic Medical Center - University of Amsterdam, Meibergdreef 9, 1105 AZ, Amsterdam, The Netherlands; 2 Department of Clinical Informatics, Bioinformatics, and Biostatistics, Academic Medical Center - University of Amsterdam, Meibergdreef 9, 1105 AZ, Amsterdam, The Netherlands

**Keywords:** List sequential sampling, Sample representativeness, *π*ps sample, Population-based observational studies

## Abstract

**Background:**

In population-based observational studies, non-participation and delayed response to the invitation to participate are complications that often arise during the recruitment of a sample. When both are not properly dealt with, the composition of the sample can be different from the desired composition. Inviting too many individuals or too few individuals from a particular subgroup could lead to unnecessary costs or decreased precision. Another problem is that there is frequently no or only partial information available about the willingness to participate. In this situation, we cannot adjust the recruitment procedure for non-participation before the recruitment period starts.

**Methods:**

We have developed an adaptive list sequential sampling method that can deal with unknown participation probabilities and delayed responses to the invitation to participate in the study. In a sequential way, we evaluate whether we should invite a person from the population or not. During this evaluation, we correct for the fact that this person could decline to participate using an estimated participation probability. We use the information from all previously invited persons to estimate the participation probabilities for the non-evaluated individuals.

**Results:**

The simulations showed that the adaptive list sequential sampling method can be used to estimate the participation probability during the recruitment period, and that it can successfully recruit a sample with a specific composition.

**Conclusions:**

The adaptive list sequential sampling method can successfully recruit a sample with a specific desired composition when we have partial or no information about the willingness to participate before we start the recruitment period and when individuals may have a delayed response to the invitation.

## Background

Population-based observational studies are frequently used to measure the prevalence of characteristics such as diseases by means of a sample from a population [1]. Two important problems that arise when a sample is recruited is that (*i*) not everyone in the population has the same willingness to participate in the study [2-6], and (*ii*) after inviting an individual, it might take some time before we receive a response.

Variation in the willingness to participate may bias the results of the study. To deal with this problem, we could invite more individuals from groups related to a low willingness to participate [7]. However, this approach requires that the participation probability per person or group is known before the sampling procedure starts. Unfortunately, this detailed knowledge on the willingness to participate among sub-groups in the population is often not available. If the willingness to participate is less than assumed we will invite too few individuals, which leads to a too small sample and a decreased precision. On the other hand, inviting too many individuals will lead to extra costs. Generally, we invite too many individuals when we underestimate the willingness to participate and there is delayed response to the invitation. In general, not accounting for delayed response will lead an unexpected number of extra individuals in the sample at the end of the recruitment period.

An example of a complex sampling problem is observed in the HELIUS study [8]. One objective of the HELIUS study is to measure ethnic inequalities in the incidence and prognosis of major diseases in the population of Amsterdam. The desired sample should have approximately 5000 individuals in each ethnic group, and should be representative for the population of Amsterdam. This is achieved by stratifying on the auxiliary variables: place of residence (spatial), age, (continuous), gender (categorical), and social economic status (categorical) available from municipal registries.

Unfortunately, it is not straightforward to implement stratification when we have a large number of auxiliary variables of mixed types [9]. In this case, too small or even empty strata might be obtained when we cross all strata from all variables. An alternative variance reduction technique, proposed by Grafström et al., is to obtain a well spread set of participants [10,11]. Basically, a set of participants is well spread when the number of participants is close to what is expected on average, for every set of auxiliary variables. Grafström et al. showed that the variance of commonly used estimators is usually low with a well spread set of participants.

In this paper, we use the list sequential method, developed by Bondesson and Thorburn [12] to obtain a well spread set of participants without replacement from a finite population. Instead of trying to cross all strata from all auxiliary variables, our approach is based on a distance function between individuals. Similar or almost similar individuals should seldom be invited both to participate in the study. In its current form, the list sequential sampling method cannot be used to recruit sets of participants for population-based observational studies because the list sequential sampling method assumes that (*i*) everyone participates in the study and that (*ii*) there is no delayed response to the invitation.

We developed approaches to correct for non-participation and delayed response to the invitation when we use a list sequential sampling method. The list sequential sampling method evaluates individuals from the population in a sequential order, and uses a random process to decide whether or not an individual should be invited to participate in the study. In this decision we have to correct for any non-participation. An approach is to weigh the probability of being invited with the (estimated) participation probability. When there is no or partial a-priori knowledge on the participation probability, we can estimate this probability during the recruitment period using the information from already invited individuals. To combine both prior information and information that is generated during the recruitment period, we developed a Bayesian approach to estimate the participation probabilities. Moreover, to deal with the delayed response to the invitation, we use the expected response of an individual when we have no answer yet.

We performed a simulation study to illustrate the performance of the adapted list sequential sampling method, when we have unknown heterogeneous participation probabilities and delayed response to the invitation.

## Methods

### Problem description

We consider a finite population **D** containing *n* individuals, where each individual *i* is described by a vector **x**_*i*_ of auxiliary variables. The auxiliary variables **x**_*i*_ are known for each individual before the recruitment period starts. Usually **x**_*i*_ is available from municipal or national person-registries. Examples of these variables are gender, age, place of residence, and social economic status. In addition to **x**_*i*_, each individual *i* has an unobserved outcome of interest *y*_*i*_. The goal of this paper is to obtain a sample of size *m* (*m*<*n*) from **D**, in which we can observe *y*_*i*_.

A sample is described by the vector **s**=(*s*_1_,…,*s*_*n*_), where *s*_*i*_ takes the value 1 if individual *i* is in the sample and 0 otherwise [13,14]. With this representation there are 2^*n*^ possible samples. Before the recruitment period starts we need to determine *π*_*i*_, which is the probability that individual *i* is included in **s** (i.e. *p*(*s*_*i*_=1)=*π*_*i*_). We want to recruit a sample of *m* individuals and therefore $\overset{n}{\underset{i=1}{\sum }}\pi_{i}=m$, where *m* is a positive integer.

Different choices can be made for the inclusion probabilities *π*_*i*_. For instance, we can assign equal inclusion probabilities to all individuals, i.e. *π*_*i*_ = *m*/*n*. In this case, the sample **s** is expected to be a ‘miniature’ version of the population **D**, because we expect **s** to have approximately the same composition of auxiliary characteristics as **D**. In this case, the sample is referred to as a representative sample [11]. However, *π*_*i*_ is frequently chosen to be proportional to **x**_*i*_. For example, by oversampling a rare subgroup we could increase the precision of the result for that particular subgroup [15].

### List sequential sampling method

To obtain the sample we use the list sequential method based on sampling without replacement developed by Bondesson and Thorburn [12]. To illustrate the list sequential method, we first consider the situation in which all invited individuals will participate in the study.

During the recruitment period, we sequentially decide for each individual *i* from **D** whether we include this individual in the sample (*s*_*i*_=1) or not (*s*_*i*_=0). After this decision, the probability of being included in the sample for the remaining non-invited individuals from **D** is updated. Let $\pi^{\left(0\right)}=\left(\pi_{1}^{\left(0\right)},\ldots ,\pi_{n}^{\left(0\right)}\right)$ be the vector of initial inclusion probabilities which is determined *before* the sampling procedure starts, i.e. $\pi_{i}^{\left(0\right)}=\pi_{i}$. We sequentially evaluate each individual *i* from the population and update the inclusion probabilities of all non-evaluated individuals after each evaluation. For the first individual, we have $p(s_{1})=\pi_{1}^{\left(0\right)}$. Depending on whether individual 1 is included in the sample or not, the inclusion probabilities of all other, non-evaluated, individuals are updated. This gives us the vector *π*^(1)^, from which we use $\pi_{2}^{\left(1\right)}$ to determine *s*_2_; i.e. decide whether to include the second individual in the sample or not. The updating scheme can be represented as

$$\begin{array}{cc} \pi^{\left(0\right)} & =\left(\pi_{1}^{\left(0\right)}\;\;\;\;\pi_{2}^{\left(0\right)}\;\pi_{3}^{\left(0\right)}\;\pi_{4}^{\left(0\right)}\;\ldots \;\pi_{n}^{\left(0\right)}\right)\\ \pi^{\left(1\right)} & =\left(s_{1}\;\;\;\;\pi_{2}^{\left(1\right)}\;\pi_{3}^{\left(1\right)}\;\pi_{4}^{\left(1\right)}\;\ldots \;\pi_{n}^{\left(1\right)}\right)\\ \pi^{\left(2\right)} & =\left(s_{1}\;\;\;\;s_{2}\;\;\;\;\;\pi_{3}^{\left(2\right)}\;\;\pi_{4}^{\left(2\right)}\;\;\ldots \;\pi_{n}^{\left(2\right)}\right)\\ \pi^{\left(3\right)} & =\left(s_{1}\;\;\;\;s_{2}\;\;\;\;\;\;s_{3}\;\;\;\;\;\;\pi_{4}^{\left(3\right)}\;\;\ldots \;\pi_{n}^{\left(3\right)}\right) \end{array}$$

Generally, when we evaluate individual *i*, we will use the inclusion probability $\pi_{i}^{\left(i-1\right)}$ to determine *s*_*i*_. After the evaluation of individual *i*, we update all probabilities $\pi_{j}^{\left(i\right)}$, for *j*>*i* with

(1) $$\pi_{j}^{\left(i\right)}=\pi_{j}^{\left(i-1\right)}-\left(s_{i}-\pi_{i}^{\left(i-1\right)}\right)w_{j-i}^{\left(i\right)},$$

where $w_{j-i}^{\left(i\right)}$ are weights that may depend on *s*_1_,*s*_2_,…,*s*_*i*−1_. Note that $w_{j-i}^{\left(i\right)}$ determines how $\pi_{j}^{\left(i\right)}$ is affected by the sampling outcome from the individual *i*, since $w_{j-i}^{\left(i\right)}$ influences the second order inclusion probability *p*(*s*_*i*_=1,*s*_*j*_=1). The sampling scheme gives a sample of size *m*, when the weights are restricted to sum up to one, i.e. $\overset{N}{\underset{j=i+1}{\sum }}w_{j-i}^{\left(i\right)}=1$. To guarantee that $0\leq \pi_{j}^{\left(i\right)}\leq 1$, all weights should satisfy

(2) $$\begin{array}{ll} - & \min\left(\frac{1-\overset{\left(i-1\right)}{\underset{j}{\pi }}}{1-\overset{\left(i-1\right)}{\underset{i}{\pi }}},\frac{\overset{\left(i-1\right)}{\underset{j}{\pi }}}{\overset{\left(i-1\right)}{\underset{i}{\pi }}}\right)\leq \overset{\left(i\right)}{\underset{j-i}{w}}\leq \\ & \min\left(\frac{\overset{\left(i-1\right)}{\underset{j}{\pi }}}{1-\overset{\left(i-1\right)}{\underset{i}{\pi }}},\frac{1-\overset{\left(i-1\right)}{\underset{j}{\pi }}}{\overset{\left(i-1\right)}{\underset{i}{\pi }}}\right) \end{array}$$

Within these bounds, we can impose different restrictions on $w_{j-i}^{\left(i\right)}$, resulting in samples with certain characteristics. Generally, when $w_{j-i}^{\left(i\right)}>0$ we have corr(*s*_*i*_=1,*s*_*j*_=1)<0 (i.e. a negative correlation between the sampling indicators of individuals *i* and *j*), whereas with $w_{j-i}^{\left(i\right)}<0$, we have corr(*s*_*i*_=1,*s*_*j*_=1)>0. For more detail about the list sequential method, we refer the reader to respectively theorem 1 and remark 1 from Bondesson and Thorburn [12].

### Well spread samples

We are interested in recruiting a well spread sample with the list sequential sampling method. Usually, a well spread sample leads to parameter estimates with low variances. Before we can introduce the definition of a well spread sample, we require the concept of coherent subsets. Let *d*(*i*,*k*) be the distance between individuals *i* and *k*. A subset **D**^′^ from the population **D** is coherent if the following holds. First, let some individual *i*∈**D**^′^. Individual *k* is included in **D**^′^ if and only if *d*(*i*,*k*)≤*r*, where *r*≥0. Consequently, **D**^′^ can be constructed by including all individuals within a ball of radius *r* around individual *i*.

Grafström and Schelin considered a sample to be well spread with respect to the inclusion probabilities ***π*** when, for every coherent subset **D**^′^⊂**D**,

(3) $$n^{\prime }\approx \underset{i\in D^{\prime }}{\sum }\pi_{i}.$$

A smaller distance to individual *i* increases the probability of being included in the coherent subset **D**^′^. To satisfy (3), it is clear that the inclusion probability of individual *i* should be more influenced by the sampling indicators *s* of individuals with a smaller distance. We propose to measure distance between individuals with the auxiliary variables **x**, where *d*(**x**_*i*_,**x**_*k*_) is the distance between individual *i* and *k*. Based on the types of auxiliary variables, we can choose, for instance, the Mahalanobis or the Manhattan distance.

To obtain a well spread sample with the list sequential sampling method, we will use preliminary weights which are specified *before* the recruitment period starts. The preliminary weight ${\tilde{w}}_{k}^{\left(i\right)}$ reflects the effect of *s*_*k*_ from individual *k* on the inclusion probability of individual *i*. The weights are referred to as preliminary because the upper bound from (2) has an effect on the conditional inclusion probabilities.

The preliminary weights are constructed in the following way. Let $c_{k}^{\left(i\right)}$ be the rank of the distance of the *k*^*t* *h*^ individual to individual *i*, where *k*≠*i*. We rank the distances in ascending order, where we assign *c*^(*i*)^=1 to the closest individual, *c*^(*i*)^=2 to the second closest individual, and so on. To construct the preliminary weights, we could use the linear function

(4) $${\tilde{w}}_{k}^{\left(i\right)}=\mu +c_{k}^{\left(i\right)}\lambda ,$$

where the weights *μ* and *λ*≤0 are arbitrarily chosen weights. The sampling indicator *s*_*k*_ of individual *k* has a larger effect on individuals at smaller distance, whereas it has less effect on individuals at further distance. To recruit a set of approximately *m* individuals, we restrict the weights to satisfy $\underset{k\neq i}{\sum }{\tilde{w}}_{k}^{\left(i\right)}=1$.

### Heterogeneous participation probabilities

A problem of sampling from population **D** is that individuals that are invited to participate in the study can decline the invitation. Let **b**=(*b*_1_,…,*b*_*n*_) be the vector that indicates whether an individual *i* is invited to participate (*b*_*i*_=1) or not (*b*_*i*_=0). When individual *i* refuses to participate in the study, we have *s*_*i*_=0 and we do not observe *y*_*i*_. Let ***ϕ*** =(*ϕ*_1_,…,*ϕ*_*n*_) be the vector that contains the participation probability per person in the population, where *ϕ*_*i*_=*p*(*s*_*i*_=1|*b*_*i*_=1). Note that when every invitee participates (i.e. *ϕ*_*i*_=1, for *i*=1,…,*n*), we have **s**=**b**.

Let ${\tilde{\pi }}_{i}^{\left(i-1\right)}$ be the inclusion probability $\pi_{i}^{\left(i-1\right)}$ corrected for non-participation, i.e. the probability of being invited to participate in the study for individual *i* from **D**. When *ϕ*_*i*_ is known before the recruitment period starts, non-participation can be dealt with by using ${\tilde{\pi }}_{i}^{\left(i-1\right)}=\pi_{i}^{\left(i-1\right)}/\phi_{i}$ as probability to invite individual *i*. Moreover, we can use the updating rule from (1) to update the inclusion probabilities of the non-evaluated individuals $\pi_{j}^{i}$, *j*>*i*, after individual *i* responded to the invitation. This will give us a sample that approximately satisfies the inclusion probabilities ***π***.

The following small sampling problem illustrates this modification. Consider that, for the first individual, we have $\pi_{1}^{\left(0\right)}=0.25$ and *ϕ*_1_=0.5. The probability to invite this individual is therefore ${\tilde{\pi }}_{1}^{\left(0\right)}=0.5$. Using this strategy there might be some individuals *i* with ${\tilde{\pi }}_{i}^{\left(i-1\right)}>1$. This means that the participation probability of individual *i* is too low with respect to $\pi_{i}^{\left(i-1\right)}$; the desired probability to be included in **s** for individual *i* cannot be reached. For instance, this would happen in the example above for individual 1 when *ϕ*_1_=0.1 and consequently ${\tilde{\pi }}_{1}^{\left(0\right)}=2.5$. This means that we have to invite individual 1 two and a half times to satisfy $\pi_{i}^{\left(0\right)}$. Because we can only invite an individual once, we restrict all values ${\tilde{\pi }}_{i}^{\left(i-1\right)}$ to be one or lower.

### Adaptive list sequential sampling method

Usually, *ϕ*_*i*_ is not known before the recruitment period starts. In this section we suggest how *ϕ*_*i*_ can be estimated adaptively during the recruitment period. In addition, we consider delayed response to the invitation.

For each individual, we have some knowledge about the willingness to participate before the recruitment period starts. For example, we might have participation estimates from a small pilot study or from previously performed studies. In addition, information from the invited individuals becomes available during the recruitment period. Therefore, we propose to use a Bayesian method to estimate the participation probability of individual *i* during the recruitment period, in which we use both the available prior knowledge and the information that becomes available during the recruitment period.

Let **z**_*i*_ be the vector of all observed characteristics of individual *i*, which are related to the participation probability. We assume a missing at random type of mechanism for the participation probabilities, where the participation probability of individual *ionly* depends on observed characteristics **z**_*i*_, i.e. *p*(*s*_*i*_=1|*b*_*i*_=1,**z**_*i*_). The participation probability can be written as

(5) $$p(s=1|b_{i}=1,z_{i},\alpha ,\beta )=\frac{\exp\{\alpha +f(z_{i},\beta )\}}{1+\exp\{\alpha +f(z_{i},\beta )\}},$$

where *α* is the intercept term, and *f*() is a function of the observed characteristics **z**_*i*_ and the regression weights ***β***. Because more information becomes available during the recruitment period, the participation probability estimates become more accurate. The vector of estimated participation probabilities of all *n* individuals *after* the evaluation of individual *i* is denoted as ${\hat{\phi }}^{\left(i\right)}=({\hat{\phi }}_{1}^{\left(i\right)},\ldots ,{\hat{\phi }}_{n}^{\left(i\right)})$. We then adapt the inclusion probabilities as ${\tilde{\pi }}_{i}^{\left(i-1\right)}=\pi_{i}^{\left(i-1\right)}/{\hat{\phi }}_{i}^{\left(i-1\right)}$.

After an invitation has been send to an individual, it might take some time to get a response. Let $u_{j}^{\left(i\right)}$ be the indicator whether individual *j* has responded to the invitation *before* individual *i* is evaluated, where $u_{j}^{\left(i\right)}=1$ when we observe *s*_*j*_ and $u_{j}^{\left(i\right)}=0$ when we do not observe the participation indicator *s*_*j*_ during the evaluation of individual *i*. Note that when individual *j* has not been invited (i.e. *b*_*j*_=0), *s*_*j*_=0 since individual *j* is not included in the set of participants. A problem of delayed response is that we cannot use the update rule from (1) to determine $\pi_{j}^{\left(i\right)}$, when the participation indicator of the previous individual is not observed. Consequently, we cannot update $\pi_{j}^{\left(i\right)}$ which means that our sampling method is less successful in recruiting a well spread sample. As a solution, we propose to use the data from *all* previously invited individuals, and replace the non-observed participation indicators with their estimated expected value. We use this approach in step 1 of the adaptive list sequential sampling method listed below.

Before we start the adaptive list sequential sampling method, we specify the vector ***π***^(0)^= ***π***, which contains the initial probabilities of being included in **s** for every individual *i* in **D**. The desired number of individuals in **s** is $m=\overset{n}{\underset{i=1}{\sum }}\pi_{i}^{\left(0\right)}$, where *m* is a positive integer. The first individual from **D** is invited with the probability ${\tilde{\pi }}_{1}^{\left(0\right)}=\pi_{1}^{\left(0\right)}/{\hat{\phi }}_{1}^{\left(0\right)}$, where ${\hat{\phi }}_{1}^{\left(0\right)}$ is an initial guess of the participation probability of the first individual. All other individuals from **D** are invited in a sequential way, where the steps of the adaptive list sequential sampling method for individual *i*=2,…,*n* are **1. Calculate**$\pi_{i}^{\left(i-1\right)}$To deal with delayed response to the invitation, we propose to use a modified version of the column-wise updating rule proposed by Bondesson and Thorburn [12]. We calculate $\pi_{i}^{\left(i-1\right)}$ by iterating over *k*=1,2,…,*i*−1, where

(6) $$\pi_{i}^{\left(k\right)}=\pi_{i}^{\left(k-1\right)}-\left(s_{k}-\pi_{k}^{\left(k-1\right)}\right)w_{k}^{\left(i\right)},$$

and $w_{k}^{\left(i\right)}$ is calculated as

$$w_{k}^{\left(i\right)}=\min\left({\tilde{w}}_{k}^{\left(i\right)},\frac{\pi_{i}^{\left(k-1\right)}}{1-\pi_{k}^{\left(k-1\right)}},\frac{1-\pi_{i}^{\left(k-1\right)}}{\pi_{k}^{\left(k-1\right)}}\right).$$

The weight $w_{k}^{\left(i\right)}$ determines the effect of *s*_*k*_ on $\pi_{i}^{\left(k\right)}$ and therefore also $\pi_{i}^{\left(i-1\right)}$. The choice of preliminary weights ${\tilde{w}}_{k}^{\left(i\right)}$ is discussed in the previous section. Because (6) still requires the *observed* indicators *s*_1_,*s*_2_,…,*s*_*i*−1_, we modify (6) to deal with delayed response to the invitation. When $u_{k}^{\left(i\right)}=0$, we replace *s*_*k*_ with its estimated expectation ${\hat{\phi }}_{k}^{\left(i-1\right)}b_{k}$, where ${\hat{\phi }}_{k}^{\left(i-1\right)}$ is the participation probability estimate of individual *k* from the previous evaluation *i*−1. The delayed response adjusted column-wise updating rule from (6) is

$$\pi_{i}^{\left(k\right)}=\left\{\begin{array}{ll} \pi_{i}^{\left(k-1\right)}-\left(s_{k}-\pi_{k}^{\left(k-1\right)}\right)w_{k}^{\left(i\right)}\; & \text{if}\;u_{k}^{\left(i\right)}=1,\\ \pi_{i}^{\left(k-1\right)}-\left(\left\{{\hat{\phi }}_{k}^{\left(i-1\right)}b_{k}\right\}-\pi_{k}^{\left(k-1\right)}\right)w_{k}^{\left(i\right)}\; & \text{if}\;u_{k}^{\left(i\right)}=0. \end{array}\right)$$

**2. Calculate**${\tilde{\pi }}_{i}^{\left(i-1\right)}$Decide whether individual *i* should be invited to participate in the study, where *b*_*i*_=1 if the individual is invited and *b*_*i*_=0 if not. This decision is based on the probability of being invited,

(7) $${\tilde{\pi }}_{i}^{\left(i-1\right)}=\frac{\pi_{i}^{\left(i-1\right)}}{{\hat{\phi }}_{i}^{\left(i-1\right)}},$$

where ${\hat{\phi }}_{i}^{\left(i-1\right)}$ is the participation probability estimated from the previous evaluation *i*−1. We draw the decision to invite individual *i* from a Bernoulli distribution with $p(b_{i}=1)={\tilde{\pi }}_{i}^{\left(i-1\right)}$. **3. Update the vector** ***ϕ***^***(i)***^ Let **R**^(*i*)^={*r*;*b*=1,*u*^(*i*)^=1,*r*∈**D**} be the set of all *m*_*i*_ individuals that responded to the invitation to participate. Each individual from **R**^(*i*)^ is described by *r*=(*s*,**z**), where *s*=1 when invitee *r* participates and *s*=0 otherwise, and **z** is a vector of known characteristics. The participation probability of individual *k* is defined as (5). Because we might have some a-priori knowledge about the intercept *α* and the regression weights ***β***, we use Bayesian inference to estimate the posterior distribution *g*(*α*, ***β*** |**R**^(*i*)^), i.e.

(8) $$g\left(\alpha ,\beta |R^{\left(i\right)}\right)=\frac{h\left(R^{\left(i\right)}|\alpha ,\beta \right)f(\alpha ,\beta |\theta )}{\int_{\left(\alpha ,\beta \right)}h(R^{\left(i\right)}|\alpha ,\beta )f\left(\alpha ,\beta |\theta \right)\partial (\alpha ,\beta )}.$$

where ***θ*** is a vector of parameters, and *f*() is the prior distribution of (*α*, ***β***). The likelihood of **R**^(*i*)^ given (*α*, ***β***) is

$$h(R^{\left(i\right)}|\alpha ,\beta )\;=\;\overset{m_{i}}{\underset{\ell =1}{\prod }}p{\left(s_{\ell }\;=\;1|z_{\ell },\alpha ,\beta \right)}^{s_{\ell }}{\left\{1-p(s_{\ell }=1|z_{\ell },\alpha ,\beta )\right\}}^{1-s_{\ell }}$$

where *p*(*s*_*ℓ*_=1|**z**_*ℓ*_,*α*, ***β***) is given by (5). Following (8) we update the vector of estimated participation probabilities ${\hat{\phi }}^{\left(i\right)}$, where for individual *k*=1,…,*n*

$${\hat{\phi }}_{k}^{\left(i\right)}=\int_{\left(\alpha ,\beta \right)}p(s_{k}=1|z_{k},\alpha ,\beta )g(\alpha ,\beta |R^{\left(i\right)})\partial (\alpha ,\beta ).$$

To estimate ${\hat{\phi }}_{k}^{\left(i\right)}$, we can use quadrature or MCMC methods. The values of ***θ*** depend on the amount of prior knowledge that is available before the recruitment period starts. For instance, we can assume that (*α*, ***β***) is sampled from some flat distribution with large variance when no prior knowledge is available.

### Simulations

We illustrated the performance of the adaptive list sequential sampling method with two simulations. In these two simulations, we created populations with *unknown* heterogeneous willingness to participate and delayed response to the invitation. The first simulation was focused on recruiting a well spread, representative set of participants. In the second simulation, we investigated stratified sampling from a population in which some subgroups were over-represented.

#### Simulation 1

Consider a population **D** of size *n*=4000 from which we drew a random sample without replacement of size *m*=400 with the adaptive list sequential sampling method. To recruit a representative sample from the population, we assigned equal inclusion probabilities to all individuals from the population; i.e. $\pi_{i}^{\left(0\right)}=m/n=0.1$ for *i*=1,…,*n*. When the sample is well spread, the distribution of the auxiliary characteristics **x** should be approximately similar in the population and the sample.

The data was generated as follows. The vector **z**_*i*_ was drawn from a multivariate normal distribution with means zero, and covariances zero. The probability of positively responding to the invitation was *p*(*s*_*i*_=1|*b*_*i*_=1,**z**_*i*_)=invlogit[ *α*+**z**_*i*_ ***β***], where invlogit denotes the inverse logit transformation, *α*=1, and ***β*** =(0.3,−0.7,0.1,0.4). The response was drawn from a Bernoulli distribution with *p*(*s*_*i*_=1|*b*_*i*_=1,**z**_*i*_). In addition, for individual *i*, delayed response to the invitation was simulated by drawing time *t*_*i*_ from a Poisson distribution with expectation 15. Individual *i* responded to the invitation *after* the evaluation of individual *i*+*t*_*i*_. Thus if *t*_*i*_=0, individual *i* responded immediately to the invitation.

For individual *i*, the characteristics **x**_*i*_ were drawn from a multivariate normal distribution with means zero, variances one, and covariance matrix

$$\left(\begin{array}{cccc} 1.00 & 0.20 & -0.50 & 0.30\\ 0.20 & 1.00 & 0.20 & -0.40\\ -0.50 & 0.20 & 1.00 & -0.20\\ 0.30 & -0.40 & -0.20 & 1.00 \end{array}\right).$$

To obtain a well spread and representative sample, we used the adaptive list sequential method. To satisfy (3), we used the Mahalanobis distance to quantify the distance between individuals. We ranked the distances in ascending order and used the order to determine the preliminary weights ${\tilde{w}}_{k}^{\left(i\right)}$, for *i*=1,…,*n* and *k*≠*i*. Using (4), we specified the following adaptive list sequential sampling methods with different characteristics ***Simple random sampling:*** Assign zero to all weights ${\tilde{w}}_{k}^{\left(i\right)}$. Consequently, $w_{k}^{\left(i\right)}=0$ and therefore $\pi_{i}^{\left(i-1\right)}=\pi_{i}^{\left(0\right)}$. With these weights, we used the initial inclusion probability $\pi_{i}^{\left(0\right)}$ to determine whether we should invite individual *i*. ***Adjusted sampling 1:*** The inclusion probability of individual $i\;\pi_{i}^{\left(i-1\right)}$ was equally influenced by all *n*−1=3999 other individuals by using the preliminary weights ${\tilde{w}}_{k}^{\left(i\right)}=1/3999$. ***Adjusted sampling 2:*** Only the 50 nearest neighbors of individual *i* influenced the inclusion probability $\pi_{i}^{\left(i-1\right)}$ by using the preliminary weights

$${\tilde{w}}_{k}^{\left(i\right)}=\left\{\begin{array}{ll} 1/50\; & \text{if}\;c_{k}\leq 50,\\ 0\; & \text{otherwise}. \end{array}\right)$$

We used an estimated participation probability to deal with non-participation. Two different approaches to estimate the participation probability were evaluated. The first approach was to use all available data to estimate the participation probability, i.e. ${\hat{\phi }}_{i}^{\left(i-1\right)}=p(s_{i}=1|b_{i}=1,z_{i})=\text{invlogit}[\;\hat{\alpha }+z_{i}\hat{\beta }]$. With the second approach, we assumed that **z**_*i*_ had no impact on the participation probability, i.e. ${\hat{\phi }}_{i}^{\left(i-1\right)}=p(s_{i}=1|b_{i}=1,z_{i})=\text{invlogit}[\;\hat{\alpha }]$. The second approach was used to investigate whether the impact of miss-specifying ${\hat{\phi }}_{i}^{\left(i-1\right)}$ had a large impact on how well the sample was spread.

We assumed that we had no prior knowledge about the participation probability before the recruitment period started. Therefore flat, non-informative priors were used for *α* and all regression weights ***β*** by assuming they followed normal distributions with means zero and variance 100. Because we assumed zero means, the initial estimated participation probabilities were 50%, i.e. ${\hat{\phi }}_{i}^{\left(0\right)}=0.5$ for *i*=1,…,*n*.

We quantified how well a sample was spread with the following measure based on Voronoi polytopes, suggested by Grafström and Lundström[10]. Let individual *i*∈**s**, i.e. individual *i* is included in the set of participants **s**. The Voronoi polytope *v*_*i*_ consists of all individuals *j* from the population **D** for which *d*(**x**_*i*_,**x**_*j*_)≤*d*(**x**_*k*_,**x**_*j*_), for all other individuals *k*∈**s**. Note that when *d*(**x**_*i*_,**x**_*j*_)=*d*(**x**_*k*_,**x**_*j*_), individual *j* is included in both polytopes *v*_*i*_ and *v*_*k*_, but weighted with 1/2.

Let *q*_*i*_ be the sum of initial inclusion probabilities of the individuals in *v*_*i*_,

$$q_{i}=\underset{j\in v_{i}}{\sum }\pi_{j}.$$

Grafström and Lundström showed that a sample can be considered to be well spread if *q*_*i*_ is one or close to one for all polytopes *v*_*i*_. Therefore, a measure to quantify how well spread a sample is

$$R=\frac{1}{n}\underset{i\in s}{\sum }{\left(q_{i}-1\right)}^{2},$$

where a low *R* corresponds to well spread sample. To investigate how well the adaptive list sequential sampling methods performed in recruiting a well spread sample, the simulation was performed 1000 times. We calculated the mean and variance of *R*, and the average sum of recruited participants. Note that the best adaptive list sequential sampling method should give us a set of approximately 400 participants with a low *R* in *every* simulation.

#### Simulation 2

In simulation 2, we considered a population **D** of size *n*=5000, in which each individual was described by a categorical auxiliary variable *x*_*i*_ and a unobserved binary outcome of interest *y*_*i*_. The auxiliary variable *x*_*i*_ had five possible values *g*. The main goal of this simulation was to estimate the sum of the outcome *y* in the population, denoted as $Y=\overset{n}{\underset{i=1}{\sum }}\;y_{i}$, with a set of participants in which we can measure *y*. Moreover, we had resources to measure *y* in a set of participants of size *m*=500. The set of participants was obtained with an adaptive list sequential sampling method where we dealt with non-participating during the recruitment period.

Individuals in different subgroups had different participation probabilities and different frequencies of the outcome *y*. The characteristics of the populations were

$$\begin{array}{rlllll} g= & (1 & 2 & 3 & 4 & 5)\\ p(x_{i}=g)= & (40\% & 20\% & 20\% & 10\% & 10\%)\\ p(s_{i}=1|b_{i}=1,x_{i}=g)= & (50\% & 60\% & 70\% & 80\% & 90\%)\\ p(y_{i}=1|x_{i}=g)= & (10\% & 20\% & 30\% & 40\% & 50\%) \end{array}$$

where *p*(*s*_*i*_=1|*b*_*i*_=1,*x*_*i*_=*g*) was the participation probability of individual *i* given *x*_*i*_=*g*, i.e. for individual *i* the probability of participating depended on *x*_*i*_. The response to an invitation was drawn from a Bernoulli distribution with probability *p*(*s*_*i*_=1|*b*_*i*_=1,*x*_*i*_=*g*). Moreover, $E(Y)=n\overset{5}{\underset{g=1}{\sum }}p(y_{i}=1|x_{i}=g)p(x_{i}=g)=1150$.

The individuals in the set of participants **s** were used to estimate *Y*, denoted as ${Ŷ}_{\text{HT}}$, where we used the Horvitz-Thompson estimator and its variance [14-16] to determine ${Ŷ}_{\text{HT}}$. The estimate ${Ŷ}_{\text{HT}}$ was calculated as

(9) $${Ŷ}_{\text{HT}}=\underset{j\in s}{\sum }\frac{y_{j}}{\pi_{i}^{\left(0\right)}}$$

where $\pi_{i}^{\left(0\right)}$ was the desired probability of being included in the set of participants **s**, specified before the recruitment period started. The variance of ${Ŷ}_{\text{HT}}$ was approximated with

$$\hat{V}({Ŷ}_{\text{HT}})=\underset{i\in s}{\sum }\underset{j\in s}{\sum }\frac{\pi_{\text{ij}}^{\left(0\right)}-\pi_{i}^{\left(0\right)}\pi_{j}^{\left(0\right)}}{\pi_{\text{ij}}^{\left(0\right)}}\frac{y_{i}}{\pi_{i}^{\left(0\right)}}\frac{y_{j}}{\pi_{j}^{\left(0\right)}}$$

where $\pi_{\text{ij}}^{\left(0\right)}$ is the second order joint-inclusion probability of the *i*^*t* *h*^ and *j*^*t* *h*^ individuals in **s**, i.e. $\pi_{\text{ij}}^{\left(0\right)}=p(s_{i}=1,s_{j}=1)$. To determine $\pi_{\text{ij}}^{\left(0\right)}$, we used the sample based approximation technique proposed by Hájek [17,18].

The set of participants **s** was obtained with the adaptive list sequential sampling method. Before the recruitment period started, we specified the vector ***π***^(0)^. We considered a vector ***π***^(0)^, in which the probability of being included in **s** was proportional to the size of group *g* in the population. Because not all groups were observed with the same frequency in **D**, we oversampled the smaller subgroups in such a way that each group *g* was observed with similar frequency in **s**. For each invited individual with *x*=1, we have to invite 2, 2, 4, and 4 individuals with respectively *x*=2,3,4,5 to obtain an equal number of individuals from each group in **s**. Therefore, depending on the value of *x*_*i*_, we used the following probabilities for individual *i*

$$\pi_{i}^{\left(0\right)}=\left\{\begin{array}{cc} 0.05\; & \text{if}\;x_{i}=1\\ 0.10\; & \text{if}\;x_{i}=2\text{or}\;x_{i}=3\\ 0.20\; & \text{if}\;x_{i}=4\text{or}\;x_{i}=5 \end{array}\right)$$

Note that we could also use stratified sampling to get our desired set of participants because we only have five disjoint groups. However when we have a large number of groups, stratification becomes impracticable. A large number of groups is no problem for the (adaptive) list sequential sampling design, if it is possible to specify a distance measure between individuals (see (3)). With ***π***^(0)^, we expected to have an equal number of individuals for each subgroup *g* in the set of participants.

We considered two adaptive list sequential methods to recruit the sample. ***Simple random sampling:*** Assign zero to all weights ${\tilde{w}}_{k}^{\left(i\right)}$. Therefore $\pi_{i}^{\left(i-1\right)}=\pi_{i}^{\left(0\right)}$. ***Adjusted sampling:*** To recruit a well spread sample, the inclusion probability of individual *i* should *only* be influenced by individuals located in the same group. Therefore, we used the following preliminary weights

$${\tilde{w}}_{k}^{\left(i\right)}=\left\{\begin{array}{ll} 1/(n_{g}-1)\; & \text{if}\;x_{i}=g\text{and}\;x_{k}=g,\\ 0\; & \text{otherwise}, \end{array}\right)$$

where *n*_*g*_ is the number of individuals in group *g*.

For both adaptive list sequential sampling methods, we used the following model to describe the participation probability

$$p(s_{i}=1|b_{i}=1,x_{i}=g,\beta )=\frac{\exp\left[\beta_{g}I(x_{i}=g)\right]}{1+\exp\left[\beta_{g}I(x_{i}=g)\right]}$$

where *β*_*g*_ is the regression weight for group *g*. Because we assumed we had no a-priori information about the participation probabilities, we used non-informative priors for ***β*** by sampling all five parameters *β*_*g*_ from a normal distribution with mean zero and variance 100. For individual *i*, delayed response to the invitation was simulated by drawing time *t*_*i*_ from a Poisson distribution with expectation 15. Individual *i* responded to the invitation *after* the evaluation of individual *i*+*t*_*i*_.

The simulations were performed 1000 times and we calculated the bias, MSE, and coverage of ${Ŷ}_{\text{HT}}$ for both adaptive list sequential methods.

## Results

### Simulation 1

The results from simulation 1 have been summarized in Table 1. The results showed that the adaptive list sequential sampling method with the adjusted sampling 2 performed best. In this approach, the participation probability of individual *i* was only influenced by the participation indicator of the 50 nearest neighbors. The recruited sets of participants better spread than with the other sampling approaches, reflected by the lower median and spread of *R*.

**Table 1:** **95% Confidence interval of *R *** **and the number of participants in simulation 1**

**Estimated participation probability:**$\text{invlogit}[\;\hat{\alpha }+z_{i}\hat{\beta }]$					
**Sampling**	**Measure** ***R***			**Number of participants**	
**method**	**2.5%**	**50%**	**97.5%**	**Mean**	**Standard deviation**
Simple random sampling	0.192	0.238	0.304	401	18
Adjusted sampling 1	0.199	0.241	0.298	397	11
Adjusted sampling 2	0.157	0.189	0.225	397	11
**Estimated participation probability:**$\text{invlogit}[\;\hat{\alpha }]$					
**Sampling**	**Measure** ***R***			**Number of participants**	
**method**	**2.5%**	**50%**	**97.5%**	**Mean**	**Standard deviation**
Simple random sampling	0.188	0.230	0.304	405	18
Adjusted sampling 1	0.197	0.238	0.291	400	11
Adjusted sampling 2	0.154	0.184	0.225	400	11

Using all the auxiliary characteristics **z**_*i*_ to estimate the participation probability of individual *i*, the simple random sampling approach resulted in a median *R* of 0.238 (95% confidence interval: 0.192–0.304). The mean number of participants with the simple random sampling approach was about 401 (95% confidence interval: 365 – 436). For the adjusted sampling 1 approach, approximately similar results were found for *R*, i.e. on average, the set of participants obtained with the simple random sampling approach and the adjusted sampling 1 approach were comparable in how well they were spread. With the adjusted sampling 1 approach, the average size of the set of participants was 397 (95% confidence interval: 376 – 418). However, compared to the simple random sampling approach, the variation in the size of the set of participants was considerably lower with the adjusted sampling 1 approach (respectively standard deviations of 18 and 11).

On average, a set of participants recruited with the adjusted sampling 2 approach was better spread than with the other two approaches. Not only was the median *R* 0.189, the spread around the median was also smaller than with the other two approaches (95% confidence interval: 0.157–0.225). The mean size of the set of participants with the adjusted sampling 2 approach was 397 (95% confidence interval: 376 – 418), which was comparable to the adjusted sampling 1 approach.

Interestingly, the performances of all three approaches remained similar when we ignored the auxiliary characteristics **z**_*i*_ in the estimation of the participation probability of individual *i*. Since fitting a model with just an intercept gave comparable results to the more complicated model where we also included **z**_*i*_, the results suggested that the adaptive list sequential sampling method was robust to miss-specification of the participation probability model.

### Simulation 2

The results from simulation 2 have been summarized in Table 2. Using the set of participants obtained with the simple random sampling approach resulted in a biased estimate of ${Ŷ}_{\text{HT}}$. With the adjusted sampling approach, ${Ŷ}_{\text{HT}}$ was more accurately estimated. This was reflected in the bias (+31 for simple random sampling and +1 for adjusted sampling), and the variance of the estimate (7995 for simple random sampling and 7817 for adjusted sampling). Consequently, the coverage of the 95% confidence interval was better when we used the adjusted sampling approach (0.86 for simple random sampling and 0.92 for adjusted sampling).

**Table 2:** **Estimated frequency **** derived from the set of participants in simulation 2**

**Sampling**	**Estimated**	**Bias**	**Variance**	**Mean squared error**	**Coverage of the**
**method**	$E({Ŷ}_{\text{HT}})$	$E(Y-{Ŷ}_{\text{HT}})$	$E[\;\hat{V}({Ŷ}_{\text{HT}})]$	$E[\;{\left({Ŷ}_{\text{HT}}-Y\right)}^{2}]$	**95% confidence interval**
Simple random sampling	1181	31	7995	14457	0.86
Adjusted sampling	1151	1	7817	10288	0.92

## Discussion

In this paper, we developed an adaptive list sequential sampling method when a random sample from the population is required and the willingness to participate varies between individuals and is not known beforehand. Our adaptive list sequential sampling method requires that the characteristics that are related to the participation probability are known of all individuals. With simulations, we showed that the adaptive list sequential sampling method could successfully deal with unknown heterogeneous participation probabilities.

In our adaptive list sequential sampling method, we evaluate each individual from the population only once. Therefore we only have one opportunity to decide whether to invite an individual or not. When we overestimate the participation probability for all individuals from the population, we end up with a too small set of participants. A simple solution for this problem would be to re-evaluate non-invited individuals until the desired size of participants in the study has been reached.

The simulations suggested that the adaptive list sequential sampling method is robust to miss-specification of the participation probability model. Just using an intercept term to describe the participation probability seems to work quite well. However, to what extent the adaptive list sequential sampling method can deal with wrong participation probability estimates was not investigated in this paper. In addition, extreme delayed response to the invitation has influence on the performance of the list sequential sampling method. Further research is necessary to determine in which situations the adaptive list sequential sampling method succeeds and fails to recruit a well spread set of participants.

A problem that was not considered here was the use of multiple invitation techniques in sampling designs. For instance, there could be individuals in the population that have a low willingness to participate when they are invited by a letter, but a much larger willingness when invited by telephone. Our method can be adopted by introducing multiple participation probabilities by extending step 3 of our algorithm and estimate multiple logistic regression participation probabilities.

## Conclusions

We showed that correcting for heterogeneity in the participation probability during the recruitment period is an effective approach when we have no or partial knowledge on the willingness to participate in population studies. By inviting individuals from the population in stages, the participation probability can be estimated and used in the sampling procedure.

## Competing interests

The authors declare that they have no competing interests.

## Authors’ contributions

All authors participated in the design of the study. MHH performed the statistical analysis and drafted the manuscript. All authors read and approved the final manuscript.

## Pre-publication history

The pre-publication history for this paper can be accessed here:

http://www.biomedcentral.com/1471-2288/14/81/prepub
